# Circulating Immune Complexes and trace elements (Copper, Iron and Selenium) as markers in oral precancer and cancer : a randomised, controlled clinical trial

**DOI:** 10.1186/1746-160X-2-33

**Published:** 2006-10-16

**Authors:** Sunali S Khanna, Freny R Karjodkar

**Affiliations:** 1 Department of Oral Medicine and Radiology, Nair Hospital Dental College, Mumbai, India; 2 Department of Oral Medicine and Radiology, Nair Hospital Dental College, Mumbai, India

## Abstract

**Aim:**

To evaluate the levels of circulating immune complexes, trace elements (copper, iron and selenium) in serum of patients with oral submucous fibrosis (OSMF), oral leukoplakia (L), and oral squamous cell carcinoma (SCC), analyze the alteration and identify the best predictors amongst these parameters for disease occurrence and progression.

**Methods:**

Circulating immune complexes (CIC) were estimated using 37.5% Polyethylene Glycol 6000(PEG) serum precipitation. Serum estimation of copper (Cu), Iron (Fe) and selenium (Se) was done using the Oxalyl Dihydrazide method, Colorimetric Dipyridyl method and the Differential Pulse Cathodic Stripping Voltametry respectively.

**Results:**

The data analysis revealed increased circulating immune complex levels in the precancer and cancer patients. Serum copper levels showed gradual increase from precancer to cancer patients. However, serum iron levels were decreased significantly in the cancer group. Selenium levels showed marked decrease in the cancer group. Among CIC, serum, copper, iron and selenium the best predictors for the occurrence of lesions were age, serum iron, CIC, serum selenium in the decreasing order.

**Conclusion:**

The present study shows that these immunological and biological markers may be associated with the pathogenesis of oral premalignant and malignant lesions and their progressions. Concerted efforts would, therefore, help in early detection, management, and monitoring the efficacy of treatment.

## Background

Oral cancer the sixth most common cancer worldwide continues to be the most prevalent cancer related to the consumption of tobacco, alcohol and other carcinogenic products[1]. While the cancer incidence remains high in South and South East Asia (its traditional high risk areas); parts of Central and Eastern Europe are seeing alarming increase and now constitute the highest incidence parts of the globe[2].

Increasing awareness on part of the providers of treatment, as well as the population in general, has led to a large proportion of patients presenting with earlier stage of the disease.

Epidemiological studies indicate that intervention at an early stage might reduce oral carcinoma related deaths. The discovery of immunological markers at a clinical, histological and molecular level has marked the end of an era of groping in the dark for clues to the basis of cancer. Significant reduction in mortality can be achieved my advances in early diagnosis and implementation of multidisciplinary treatment programmes leading to improvement of survivorship and better quality of life.

### Oral precancer and cancer

In India, oral cancer is prevalent in most areas where tobacco related practices are observed. For development of oral cancer, tobacco is the single greatest risk factor. This is due to higher concentration of carcinogenic exposure and failure to clean the carcinogens from the mucosal surface. If one observes the mouths of heavy tobacco users, the accumulation of tobacco residue may be correlated with areas of the oral cavity involved [3]. Alcohol, viruses, genetic mechanisms, candida, chronic irritation and diet deficiency states are also implicated in the etiology[4,5].

The development of oral cancer is a multistep process arising from pre-existing potentially malignant lesions. Leukoplakia is the most common precancer representing 85% of such lesions[6]. Histologically, over 95% of oral cancers are squamous cell carcinomas[7,8]. It has been suggested that a vast majority of oral squamous cell carcinomas in India arise from pre- existing Leukoplakia[9].

Likewise, the incidence of oral submucous fibrosis (OSMF) is increasing like an epidemic, targeting the younger generation. The etiology for OSMF is still obscure and a varied number of factors have been proposed. Of these, areca nut use is the most important and persistent finding in history taking[10].

### Role of circulating immune complexes

Intensive studies have documented the role of immune complexes as modulators of both cellular and humoral immune response. The occurrence of circulating immune complexes (CIC) as a marker for tumor burden and prognosis in the sera of patients with oral precancer and cancer is now well established. Recent advances in the fields of CIC, tumor progression, drug resistance, tumor cell heterogeneity and metastasis have resulted in a renewed interest in the development of non- specific immunotherapeutic modalities [11].

The overall consensus is that only a small percentage of the detected CIC in vivo represent tumor associated antigens complexed with antibodies. The bulk of CIC most likely represent auto antibodies or the reaction to denatured self proteins, microbes, normal lymphocyte, antigens and nuclear antigens[12]. Antigenic make up of CIC in cancer patients reflects the host's immune response to a variety of often overlapping antigenic stimuli and hence paves way for further studies[13].

Trace elements have been extensively studied in recent years to assess whether they have any modifying effects in the etiology of cancer. Copper, iron and selenium are essential for numerous enzymes and therefore it is reasonable to assume that variations in serum level of these biochemical markers maybe associated with the pathogenesis of oral cancer. The importance of these elements in cancer was reported by Schwartz [14] which opened the door for new diagnostic and therapeutic endeavours in many areas of medicine and specifically in the areas of oncology. Immunological and biochemical alterations in the serum of such patients can help not only in the early diagnosis, appropriate treatment but also as indicators of prognosis, as the disease progresses.

## Materials and methods

This study was carried out in Nair Hospital Dental College, Mumbai in association with Bhabha Atomic Research Centre and Topiwala National Medical College, Mumbai.

Thirty patients with (OSMF/L and 30 patients with OSCC with histopathologically proven lesions were included in this study. For comparison thirty normal subjects were also selected. The age group of these patients ranged from 25–70 years. The symptoms and signs of the patients were evaluated, after through history taking [15-18].

The following investigations were carried out in Serum obtained from 10 ml of various blood collected from the subjects -

1) Serum CICs were determined using 3.75% Polyethylene Glycol – 6000 (PEG) serum precipitation[19].

2) Serum levels of Copper (Cu) were determined using the Colorimetric Oxalyl Dihydrazide method[20].

3) Serum analysis of Iron (Fe) was done using colorimetric Dipyridyl method[20].

4) Differential Pulse Cathodic Stripping Voltametry to determine serum selenium (Se) [21].

### Statistical methods

The data was subjected to statistical analysis using the Chi Square Test, Standard Deviation, Student's unpaired t-test, correlation, ANOVA and Linear Regression.

## Results

Firstly, groupwise comparison of gender among all cases was considered. In the precancer (oral submucous fibrosis/leukoplakia) group, females were 16.70% and males formed 83.30% of the subjects. In the cancer group females formed 40% and males attributed to 60% of the subjects. In comparison with normals, the difference in gender between the three groups was not found to be statistically significant; (p value was 0.058) indicating that the 3 groups are comparable on the basis of gender (Table No. 1)

**Table No. 1:** Groupwise comparison of various variables among all cases.

**Variables**	**ANOVA test applied**		
	
	**F-value**	**P-value**	**Difference is-**
**Age**	45.073	1.10E-13	**Significant**
**CIC**	20.885	5.67E-08	**Significant**
**Cu**	4.662	0.012	**Significant**
**Fe**	78.805	2.35E-19	**Significant**
**Se**	1.714	0.187	Not significant

Most of the individuals in the study were males who had tobacco, areca nut chewing and associated habits.

The age (in years) range of the patients with precancerous condition/lesion was 34.10 in the precancer group as compared to 53.97 in cancer group and 33.65 in the normal group. The mean age in precancer and cancer group was higher than normal and the difference was statistically significant {p value1.10E-13 (1.10 × 10^-13^)}

The mean CIC levels were 0.07, 0.10 and 0.03 OD_450 _in the precancer, cancer and normal group respectively. There was a marked increased in the precancer and cancer patients. The p value 5.67 E-08 which was statistically significant (Table No. 1 Figure No 1 and 4).

**Figure 1:**
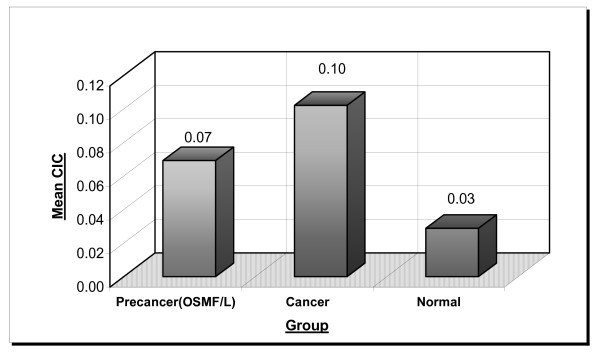
Illustrates marked increase in levels of CIC in precancer (OSMF/L) and cancer groups. as comapred to normals.

**Figure 4:**
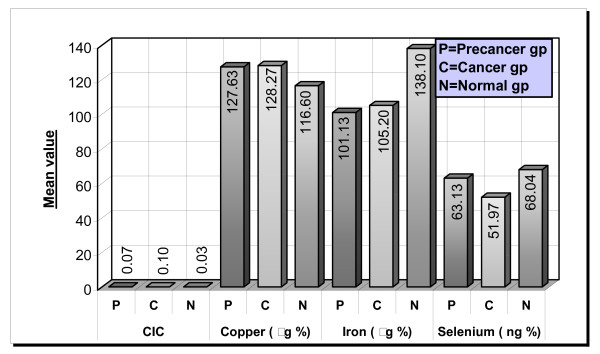
Groupwise comparison of CIC, copper, iron and selenium.

The mean serum copper levels are 127.63, 128.27 and 116.60 μg/100 ml in the precancer, cancer and normal group respectively. The p value was 0.012 which is statistically significant (Table No 1, Figure No 2 and 4)

**Figure 2:**
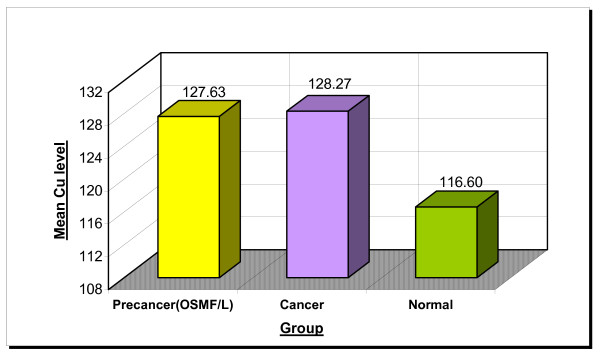
Gradual increase of copper levels from precancer to cancer as compared to normals.

The mean serum iron levels are 101.13, 105.20 and 138.10 μg/100 ml in precancer, cancer and normal groups respectively. The difference between the three groups was found to be statistically significant (p value was 2.35E-19) (Table No 1, Figure No 3 and 4)

**Figure 3:**
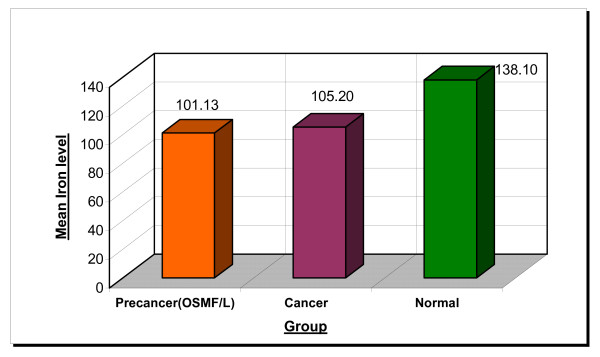
Indicates statistically significant reduction in the serum iron levels of precancer and cancer group as compared to normals.

The mean serum selenium content is 63.13, 51.97 and 68.04 ng/ml in precancer, cancer and normal groups respectively. It is significantly decreased in the cancer groups (p value was 2.35E-19) (Table No 1 and Figure No 4)

Correlation among the CIC and serum copper(Figure No 5) copper and iron, CIC and age was found to be significant in the precancer group.

**Figure 5:**
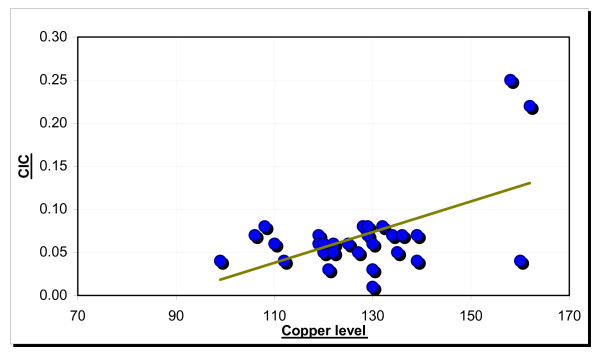
Correlation between CIC and copper in the precancer group.

Correlation among the CIC and serum copper (Figure No. 6) and serum copper and age was found to be significant in the cancer group. They showed a steady rise

**Figure 6:**
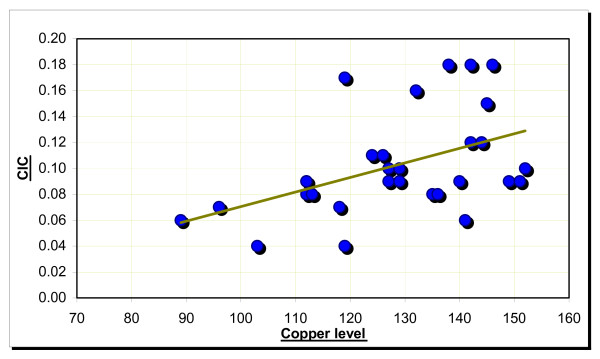
Correlation between CIC and copper in the cancer group.

Among age, CIC, serum copper, iron and selenium the best predictors for the occurrence of lesion were age, serum iron, CIC and serum selenium in the decreasing order (Figure No. 7)

**Figure 7:**
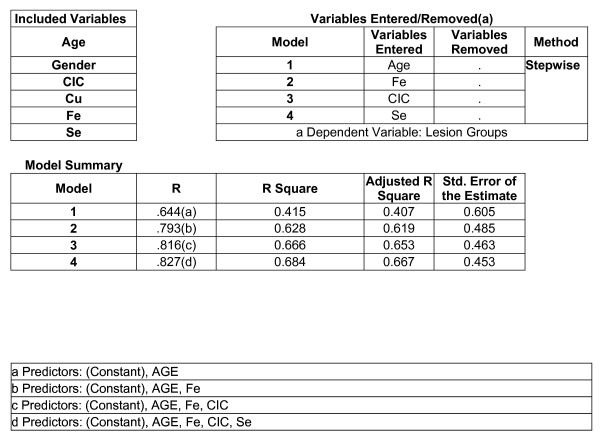
Linear Regression Analysis with type of lesions as dependant variable.

## Discussion

Research emphasizes the development of generalizations, principles or theories that will be helpful in the prediction of future occurrences.

We would all agree that no aspect of total patient care has been more important than the modern concepts of prevention, diagnosis, treatment and their systemic relationship.

The rate at which oral precancerous and cancerous lesions are spreading like an epidemic is alarming. The prevalence of oral precancerous lesions is much higher than that of oral cancer and these lesions provide useful clinical markers for oral cancer.

Immunological and biochemical alterations in the sera of such patients can help not only in early diagnosis, appropriate treatment but also as indicators of prognosis, as the disease progresses.

Oral cancer is an extremely deadly disease. It comprises approximately 2% of the total malignant tumors in Western Europe and North America, but in India, upto half of the cancers may be present in the mouth [22].

The etiology of oral squamous cell carcinomas include various carcinogens in tobacco and related products such as polynuclear aromatic hydrocarbons, and nitrosamines. Alcohol, viruses, genetic mechanisms, candida, chronic irritation and diet deficiency states rare also implicated [23,24].

Amongst the various precancerous lesions and conditions known oral submucous fibrosis is gaining importance because of the large number of case reported in the recent years in the younger generation and because of its obscure etiology. The incidence of malignant changes in patients with oral submucous fibrosis ranges from 3 to 6%. Several factors such as chillies consumption, nutritional deficiency, areca nut chewing, genetic susceptibility, autoimmunity and collagen disorders have suggested to be involved in the pathogenesis of this condition. Currently, areca nut chewing is considered to be most important etiologic factor of oral submucous fibrosis [25].

The precancerous nature of the most common of chronic oral mucosal lesions, leukoplakia is much better understood now than at any time, since it was first brought to professional attention by Sir James Paget 143 years ago. Oral leukoplakia is well established as one of the very best examples of premalignancy in man. The range of the rate of malignant transformation of this lesion is 3% to 20% [26].

The immunological abnormalities in patients with cancer in the head and neck appear to be more profound than those associated with cancers of the bronchus, breast, cervix, colon or bladder (Litchenstein et al) [27]. The immunoglobulin deposits may represent immune (antigen-antibody) complexes, since circulating immune complexes have been detected in 75% of patients with head and neck carcinoma (Scully et al) [28].

Majority of our study group consisted of males (66.67%) who had tobacco, areca nut chewing and associated habits. The mean age was higher in the patients suffering from oral carcinoma.

Gross et al [29] reported that ageing is associated with a decline in the cell mediated immunity which might predispose to oncogenesis. Circulating immune complexes have been implicated in autoimmune diseases, neoplastic diseases, infectious diseases caused by bacteria, viruses and parasites. Scully C, Barkas T. et al [30] evaluated the circulating immune complexes in patients with squamous cell carcinoma and found them significantly raised. Hoffken et al [31] concluded that the elevation of circulating immune complexes was attributed to change in the levels of complement fixing and non-complement fixing of tumour specific antibodies. This implied that it may be possible to monitor the malignant transformation of premalignant lesions. Also, emphasis should be laid on the detection of the antigenic component of the circulating immune complexes.

Chatterjee R. and Guha [32] estimated levels of circulating immune complexes using polyethylene glycol precipitation assay; which they found to be appropriate and concluded that 60% of patients with carcinoma of the buccal mucosa had markedly higher amount of immune complexes. They also noted that the amount of immune complexes present in patient's sera showed no correlation with serum level of IgG, IgA and IgM.

Balaram P et al [33] reported increased levels of circulating immune complexes in oral submucous fibrosis patients.

In the presence study the levels of CIC show a gradual increase in the precancer group and the cancer group is characterized by a marked increase in levels which was statistically significant. From these results it can be hypothetised that CIC represent the host's physiological and immunological defense response in eliciting specific antibodies upon exposure to most antigenic substances.

CIC deposition further leads to inflammation and tissue/cell damage. It also leads to suppression of cell mediated immunity and modulates the humoral response.

Circulating immune complexes are normally removed by the mononuclear phagocytic cells. However, circulating immune complexes formations or their defective clearance under certain circumstances becomes detrimental to the host, resulting in pathological deposition. Thus, altering the host immunological response leading to inflammation and tissue injury [22].

High levels of copper in areca nut, a major etiological factor in OSMF plays an initiating role in stimulation of fibrogenesis by up regulation of lysyl oxidase (Ma. R. H. et al) [32] and thereby causing inhibition of degradation of collagen. The rise in serum copper may be due to increased turnover of ceruloplasmin (a copper carrying globulin with essential oxidase activity) (Jaydeep et al) [33] in the serum of carcinoma patients. Varghese et al [34] concluded a significant reduction in serum copper in oral cancer, OSMF and leukoplakia patients.

Margalith et al [35] suggested that role of copper ions in biological damage is caused by superoxide radicals or other reducing agents such as ascorbate, which reduce the copper complex. These complexes react with hydrogen peroxide to form hydroxyl radicals that cause damage to protein, RNA and DNA that are not repairable by cellular mechanisms thus initiating the malignant process....

In this study, Serum levels of copper showed gradual increase from precancer to the cancer group as compared to normals which was statistically significant.

Serum Iron levels are considered as biochemical indicators for nutritional assessment. Utilization of iron in collagen synthesis [36] by the hydroxylation of proline and lysine leads to decreased serum iron levels in OSMF patients. In most cases clinical anemia may be a contributing factor. (Ramanathan et al) [37].

Occurrence of iron deficiency is known to present in oral cancer. Iron is known to play a key role in the development of hepatic fibrosis probably via oxidative stress and lipid peroxidation [38]. Iron is also required for collagen synthesis by enzymes in hydroxylation of proline and lysine. This hydroxylation of proline and lysine is catalyzed by proline hydroxylase and peptidyl lysine hydroxylase respectively. Peptidyl proline hydroxylase requires as co-factory molecular oxygen, ferrous iron, alpha-ketoglutarate and ascorbic acid [39].

A statistically significant reduction in the serum iron level was present in the precancer group in our study. A decrease in the iron levels in the cancer group, but higher than that of pre cancer groups was found to be significant.

Recently, haematological abnormalities in oral leukoplakia was reported by Chellacombe [39]. It was reported that poor correlation between iron indices, tumour parameters, serum iron and hemoglobin was probably due to utilization of iron by bone marrow and tumours. Ramanathan K [37] reported that oral submucous fibrosis may be the manifestation of chronic iron deficiency anemia.

There appears to be an association between the serum iron content and oral carcinogenesis. More detailed studies on a large data base should be instituted to elucidate the exact role of iron.

Selenium forms the integral part of the enzyme glutathione peroxidase, type I iodothyronine deiodinase, metalloprotein, fatty acid binding protein and selenoprotein P. therefore selenium is considered as an antioxidant nutrient and the diseases where low selenium is implicated range from nutritional disorders like protein energy malnutrition to degenerative diseases such as cancer [40].

Rajendran R [41] estimated the levels of cadmium, selenium, chromium, magnesium and calcium in the sera of patients with oral leukoplakia, oral submucous fibrosis, squamous cell carcinoma using atomic absorption spectrophotometry. In oral leukoplakia, significant decrease in the serum selenium level was reported. Also oral cancer patients showed reduced levels of selenium.

Krishnaswamy et al [42] reported decreased selenium levels in both oral/oropharyngeal cancer as compared to matched controls. Since patients in their study were at an early stage of diagnosis, they suggested low selenium level as a causative agent rather than a result of the disease.

Vijaykumar T [43] reported an increase in serum selenium in oral leukoplakia and oral cancer. Various epidemiological studies have implicated selenium as a cancer protective agent. Studies indicate that higher dietary intake of selenium in humans may be protective.

The serum selenium concentration was found to be decreased. The role of selenium is thus complex which can be attributed to its protective antioxidant role.

A significant positive correlation as present between the serum circulating immune complexes levels and copper in the precancer group. Both parameters showed a steady increase. There was a significant positive correlation found between age of subjects and circulating immune complexes, serum copper and iron levels in the cancer group

Linear regression estimates the coefficient of the linear equation involving one or more independent variables that best predict the value of the dependent variable. Applying linear regression analysis with type of lesions as dependent variable, we identified age, serum iron, CIC and serum levels of selenium as best predictors for the occurrence and progression of lesions in the decreasing order. However, gender and serum copper failed to show any predictive value for the type of lesion. Estimation of CIC and trace elements might help in early detection, differential diagnosis and treatment planning of oral premalignant and malignant lesions.

## Conclusion

The present study highlights that circulating immune complexes represent the host's physiological and immunological response in eliciting specific antibodies upon exposure to most antigenic substance.

High levels of copper in areca nuts, a major etiological factor in OSMF plays an initiating role in stimulation of fibrinogenesis by up regulation of lysyl oxidase and thereby causing inhibition of degradation of collagen and causing its accumulation thereby causing OSMF. The rise in serum copper may be due to increased turn over of ceruloplasmin in the serum of carcinoma patients.

Serum iron levels are considered as biochemical indicators for nutritional assessment. Utilization of iron in collagen synthesis by the hydroxylation of proline and lysine leads to decrease serum iron levels in OSMF patients. In most cases clinical anemia may be a contributing factor. Inadequate intake of food due to burning sensation and vesiculation in the oral cavity might also be an important factor. Reduction in the serum iron level may be due to malnutrition caused by the tumor burden in cancer patients.

A decrease in the serum selenium level in oral carcinoma patients can be attributed to the protective antioxidant role in cancer. No similar study has been done on serum levels of circulating immune complexes, trace elements, (copper, iron and selenium) as a combination in oral precancer and cancer.

An attempt was made to assess these parameters as predictors for disease occurrence and progression. We identified age, serum iron, CIC and serum levels of selenium as best predictors for the occurrence and progression of lesions in the decreasing order.

It can be suggested that immunological and biochemical assessment of oral precancer and cancer patients may help in earlier diagnosis and/or prognosis of these lesions. This may also serve in predicting malignant potential of the pre malignant lesions.

These efforts maybe of value for proactive intervention of high risk groups. (potentially malignant conditions and lesions)

Proactive intervention might be an inconvenience,

But the decision is ours,

An inconvenience rightly considered,

Or a convenience wrongly considered.

## Authors' contributions

Dr. Sunali Khanna-Study concept and design, Clinical sample and data collection, Analysis and interpretation of data, Drafting of manuscript.

Dr. Freny Karjodkar-Critical revision of manuscript, Administrative and material support and Overall supervision
